# Case of a Remarkable Abdominal Tumour in a Woman, from Which Hair and Teeth Were Extracted

**Published:** 1816-10

**Authors:** 

**Affiliations:** Licenciate of the University of Frankfort and the College of Berlin, and Physician at Hamburgh


### *Case of a remarkable Abdominal Tumour in a Woman, from which Hair and Teeth were extracted.*

[Communicated by Dr. Von Embden, Licenciate of the University of Frankfort and the College of Berlin, and Physician at Hamburgh.]

A WOMAN, at Ebingen, 37 years of age, was safely delivered in October 1814, of her eighth child, which lived a fortnight; but her abdomen remained swelled, and she continued in ill health. Four weeks after her delivery, a swelling appeared in the umbilical region, which daily increased, until it reached the size of the head of a child two years old. The swelling now sank a little, and a fluctuation was felt in it, at the distance of about an inch and a half from the navel. An opening being made with the lancet, about a pound of purulent matter was discharged, and lumps of fat and a few hairs appeared in the wound. On the 24th of March, 1815, a large bunch of hair protruded from the sore, and on the 27th, a few teeth were pulled out; a solid body, moveable backwards and forwards by the probe, remaining behind: but it vanished gradually during the process of continued suppuration. The wound then healed up; in July it was entirely closed, and in October the woman was in perfect health.

The hair, which was sent to the pathological museum at Tubingen, is of a light chesnut colour, from two to three inches long, exceedingly soft, lightly felted, without proper roots, bearing greater resemblance to very soft hair of the head, than to that of the pudenda. One of the two teeth, which resembles a child's canine tooth, rests on an irregular piece of bone; the other, which in front resembles a middle incisor, milk-tooth, entirely deviates from the shape of a human tooth, having a thin conical prong, which, as it were, forms the posterior part of the crown.

This proves that these hairs and teeth could not be the remains of a suppurated extra-uterine fœtus, but that they bear a striking analogy to the hair, bones, and teeth, which are sometimes met with in degenerated ovaria.

It is remarkable in this case, that nature was self sufficient to free itself of this mock organization; and it appears to support the opinion of Vander Haar, that degeneration of the ovaria is not so incontroulable by surgery, as it is by physic.

Hamburgh, July 6, 1816.

